# EEG-based biomarkers for optimizing deep brain stimulation contact configuration in Parkinson’s disease

**DOI:** 10.3389/fnins.2023.1275728

**Published:** 2023-10-05

**Authors:** Jana Peeters, Tine Van Bogaert, Alexandra Boogers, Till Anselm Dembek, Robin Gransier, Jan Wouters, Wim Vandenberghe, Philippe De Vloo, Bart Nuttin, Myles Mc Laughlin

**Affiliations:** ^1^Experimental Oto-rhino-laryngology, Department of Neurosciences, KU Leuven, Leuven, Belgium; ^2^Department of Neurology, University Hospitals Leuven, Leuven, Belgium; ^3^Department of Neurology, Faculty of Medicine, University of Cologne, Cologne, Germany; ^4^Laboratory for Parkinson Research, Department of Neurosciences, KU Leuven, Leuven, Belgium; ^5^Experimental Neurosurgery and Neuroanatomy, Department of Neurosciences, KU Leuven, Leuven, Belgium; ^6^Department of Neurosurgery, University Hospitals Leuven, Leuven, Belgium

**Keywords:** evoked potentials, deep brain stimulation, movement disorders, Parkinson’s disease, programming

## Abstract

**Objective:**

Subthalamic deep brain stimulation (STN-DBS) is a neurosurgical therapy to treat Parkinson’s disease (PD). Optimal therapeutic outcomes are not achieved in all patients due to increased DBS technological complexity; programming time constraints; and delayed clinical response of some symptoms. To streamline the programming process, biomarkers could be used to accurately predict the most effective stimulation configuration. Therefore, we investigated if DBS-evoked potentials (EPs) combined with imaging to perform prediction analyses could predict the best contact configuration.

**Methods:**

In 10 patients, EPs were recorded in response to stimulation at 10 Hz for 50 s on each DBS-contact. In two patients, we recorded from both hemispheres, resulting in recordings from a total of 12 hemispheres. A monopolar review was performed by stimulating on each contact and measuring the therapeutic window. CT and MRI data were collected. Prediction models were created to assess how well the EPs and imaging could predict the best contact configuration.

**Results:**

EPs at 3 ms and at 10 ms were recorded. The prediction models showed that EPs can be combined with imaging data to predict the best contact configuration and hence, significantly outperformed random contact selection during a monopolar review.

**Conclusion:**

EPs can predict the best contact configuration. Ultimately, these prediction tools could be implemented into daily practice to ease the DBS programming of PD patients.

## 1. Introduction

Deep brain stimulation (DBS) is an effective neurosurgical therapy for Parkinson’s disease (PD), where a lead is implanted into the subthalamic nucleus (STN) or the internal globus pallidus (GPi), to deliver electrical stimulation *via* an implanted neurostimulator (Benabid et al., 1991; Limousin et al., 1998; Coenen et al., 2008; Kalia et al., 2013; Schuepbach et al., 2013). Initial programming can be based on electrophysiological recordings made from the leads during implantation surgery (Chen et al., 2022; Darcy et al., 2022; Hirschmann et al., 2022; Shah et al., 2022). Programming is often guided by a monopolar review assessment where the best therapeutic DBS-contact is identified *via* systematic evaluation of the clinical response (e.g., rigidity assessment of wrist contralateral of stimulated hemisphere) elicited when stimulating on each contact separately as cathode with the anode on the implantable pulse generator. After a monopolar assessment, the programmer has a clinical response threshold (CRT), side effect threshold (SET) and a therapeutic window (TW) for each contact, where CRT is the stimulation intensity at which clinical effects can be observed. The SET is the stimulation intensity at which nontransient side effects are observed. Lastly, the TW is the difference between SET and CRT. Often, the programmer will select the contact with the widest TW to initiate programming.

Even with accurate lead positioning during surgery, programming is time-consuming, depends highly on programmer expertise, and can be exhausting for the patient. With the advent of directional leads and multiple independent current-controlled (MICC) devices, the programming parameter space has increased tremendously, leading to reduction in side effects but also to increased programming time and complexity (Ten Brinke et al., 2018; Vitek et al., 2020). Additionally, a recent study now showed that the coverages of DBS for PD are rapidly increasing and are predicted to keep increasing in the future (Meng et al., 2023).

A more objective data-driven DBS programming strategy could help solve this problem. One approach is the use of imaging, where one can visualize the lead in reference to its surrounding nuclei. Imaging is also used to visualize the spread of electrical stimulation induced by DBS, such as the electric field (EF), to guide programming (Hemm et al., 2005; Åström et al., 2009; Nguyen et al., 2019; Lange et al., 2021). When pooling individual patient data, one can create so-called clinical ‘sweet spots’, which are probabilistic stimulation maps predictive of good motor outcomes (Dembek et al., 2019) and are already being used to guide programming (Phibbs et al., 2014; Nordenström et al., 2022).

Alternatively, the use of EEG-based evoked potentials (EPs) to guide DBS programming could complement other electrophysiological methods as well as imaging-based approaches. We previously showed that EP amplitudes are significantly affected by stimulation direction and depth, and that a short-latency peak at three milliseconds (P3) is correlated to the distance to dorsolateral STN, while a long-latency peak at ten milliseconds (P10) is correlated to the substantia nigra (Peeters et al., 2023a). In a follow up study, we showed that these EPs can be used to predict clinical outcomes assessed using a monopolar review, complementary to existing imaging approaches (Peeters et al., 2023b).

In a recent study, Shah et al. (2022), reported on the use of STN local field potential (LFP) recordings and imaging data to predict the best therapeutic contact to stimulate a DBS patient using a contact prediction analysis. They concluded that the model prediction approach using features derived from LFP recordings could be a useful way to optimize DBS programming in PD patients. Based on these analyses, we now performed an additional analysis using the dataset containing EEG-based EPs and imaging data reported on in Peeters et al. (2023b) to construct a prediction model and then we performed a similar prediction analysis to investigate if EEG-derived biomarkers could be used to objectively program DBS patients.

## 2. Materials and methods

### 2.1. Patients

PD patients who met the ‘UK PD Society Brain Bank Clinical Diagnostic Criteria’ and had undergone STN-DBS surgery and were implanted with directional leads [Vercise Cartesia®, Boston Scientific (BSC, Valencia, CA, USA)] at least 3 months prior to enrollment were included in the study (Gibb and Lees, 1988; Broggi et al., 2003). All patients provided oral and written informed consent. The study was approved by the Ethics Committee Research UZ/KU Leuven (S62373) and was registered on ClinicalTrials.gov (NCT04658641). The study was conducted in accordance with the Declaration of Helsinki, the Belgian law of May 7th, 2004 on experiments on the human person and in agreement with Good Clinical Practice guidelines.

### 2.2. DBS stimulation during EEG recordings and monopolar review assessment

Patients were asked to refrain from PD medication intake overnight. One hemisphere was tested while stimulation in the other was off. First, the highest intensity that did not induce side effects was determined when stimulating on the clinical contact configuration (monopolar cathodic pulse, with anode on the case, 130 Hz frequency and 60 μs pulse width). Then, one DBS-contact was randomly selected^1^ and the frequency was decreased to a frequency of 10 Hz to enable EP recordings up to 100 milliseconds following each pulse (monopolar cathodic pulse, with the return electrode on the case and 60 μs pulse width). EPs were recorded at 10 Hz for 50 s (yielding a total of 500 epochs) at the highest intensity that did not induce side effects as defined on the clinical contact configuration. Every DBS-contact was stimulated separately, as well as the segmented contacts in ring mode. A 64-channel Active-Two BioSemi system (Amsterdam, the Netherlands) was used for all recordings (16,384 Hz sample rate, 3,200 Hz cut-off frequency low-pass filter), which were referenced to the vertex EEG channel. Three additional EEG channels were added: one on the skin over the implantable pulse generator to serve as the trigger channel for EP alignment and two on the left and right mastoid to record the stimulation artifact, on which the artifact template was based, used to reduce the stimulation artifact. Details on the artifact reduction method and preprocessing pipeline can be found elsewhere (Peeters et al., 2023a).

At least one month after the EEG recordings, patients were invited back for a double-blinded monopolar review, where both the patient and the clinician performing clinical evaluations were blinded to the contact used for stimulation while the programmer was responsible for the changes in the DBS system. The monopolar review session was always performed by the same clinician and programmer throughout the study. The clinical response threshold (CRT) was defined as the stimulation intensity that alleviated rigidity in the contralateral wrist and the side effect threshold (SET) was defined as the intensity where side effects started to appear. Rigidity was assessed according to the Movement Disorders Society Unified Parkinson’s Disease Rating Scale (part III – Section 3.3) (Heldman et al., 2011). The therapeutic window (TW) was then defined as the difference between SET and CRT. Of note, the correlation between this monopolar review data and the EP data in this patient cohort has already been published elsewhere (Peeters et al., 2023b). The monopolar review was assessed at a frequency of 130 Hz, a pulse width of 60 μs for every DBS-contact separately, as well as the segmented contacts in ring mode. The intensity was increased in steps of 0.5 mA.

### 2.3. Imaging

Lead-DBS, an open-source image processing pipeline (version 2.5.3, Berlin, Germany) (Gross et al., 2006; Horn et al., 2018) was used for postoperative lead reconstruction analysis (lead position and orientation). We used a published neuroanatomical sweet spot (motor improvement) approach for all imaging analyses (Dembek et al., 2019). We calculated the electric field (EF) at stimulation intensities of 1 mA for each DBS-contact using FastField (Baniasadi et al., 2020). We then calculated the sweet spot overlap of the EF by multiplying the electric field with the binary mask of the neuroanatomical sweet spot (centers the dorsolateral STN and covered dorsal parts of both sensorimotor STN and associative STN and surrounding white matter) (Dembek et al., 2019) and then summed all EF values that lay inside the sweet spot.

### 2.4. Contact prediction analysis

MATLAB 2022a (Mathworks Natick, MA, USA) was used for all processing and statistical analyses. Details on EP processing can be found in a previous publication (Peeters et al., 2023a). To investigate if selecting a contact based on EEG information [i.e., amplitudes of a 3-millisecond peak (P3) and a 10-millisecond peak (P10)], imaging information (i.e., EF sweet spot overlap) or combined EEG- and imaging information can fasten the process of selecting a contact based on a monopolar review assessment, we calculated contact predictions using a leave-one-out analysis. For EEG-based contact prediction, we used linear mixed models to predict the relationship between P3 and P10 peaks and clinical measures (i.e., TW, CRT, and SET). For imaging-based contact prediction, we used linear mixed models to predict the relationship between the EF sweet spot overlap to TW, CRT, and SET. Finally, for EEG- and imaging-based contact prediction, we used linear mixed models to predict the relationship between both P3 and P10 peaks, and the EF sweet spot overlap to TW, CRT, and SET. Each time, one hemisphere was left-out of the linear mixed model calculation and the TW, CRT, and SET predictions were calculated using data from the left-out hemisphere. The distribution of the linear mixed model residuals were investigated as a validation tool for these linear mixed models and were found to be distributed normally (not shown). Furthermore, the contact predictions for TW, CRT, and SET were based on either EEG data or imaging data, or a combination thereof, were also investigated per hemisphere to evaluate the contact ranking per hemisphere included in the study.

Next, the predictions from the models were compared to the true CRT, SET and TW measured during the monopolar review. To do this, we first ranked the contacts based on the predictions from the leave-one-out analysis described above from best (i.e., widest predicted TW, lowest predicted CRT, highest predicted SET) to worst (i.e., narrowest predicted TW, highest predicted CRT, lowest predicted SET) for each individual hemisphere. Based on the model ranking, we then selected the top one ranked contact and calculated the probability that the ‘truly’ best contact, based on the clinical monopolar review outcome, was among this selection. Thereafter, we selected the top two ranked contacts and calculated the probability that the truly best contact matched this selection. We repeated this procedure including incrementally more contacts until all contacts were included in the selection. The final selection with all contacts gives by definition a 100% chance of the truly best contact being included in the selection. This is termed the cumulative chance of predicting the best contact. Lastly, the area under the curve (AUC) was calculated to quantify if either EEG-based information or imaging-based information, or combined EEG- and imaging-based information could predict the best contact configuration for DBS programming with values varying between 0 (no predictive value of model) and 1 (perfect predictive value of model). As mentioned above, we also investigated the contact performance in predicting the contact with the widest TW, lowest CRT and highest SET ranked from best to worst per hemisphere based on the three above described models. For this, we calculated the normalized mean EEG-based contact prediction. A higher rank are hypothesized to indicate a contact with a wider TW, a low stimulation intensity for CRT and a high stimulation intensity for SET.

## 3. Results

Ten PD patients were included in the study. In two patients, we recorded from both hemispheres, yielding 12 tested hemispheres in total. The age, gender, dominant hemibody, levodopa equivalent daily dose (LEDD), disease duration and relevant study-related data are summarized in Table 1.

**Table 1:** Demographic data and stimulation parameters.

Hemisphere no.	Gender/age (years)	PD dominant hemibody	LEDD (in mg) at time of EEG experiment	Disease duration (in years) at time of EEG experiment	Time (in months) since DBS surgery at time of EEG experiment	Stimulation intensity (mA) at EEG experiment	Time (in months) between EEG and monopolar review
1R	F/50	R	500	10	8	6.0	10
1L	F/50	R	500	11	17	4.0	2
2L	M/55	R	430	9	7	5.0	18
3L	F/58	L	180	8	4	3.0	18
4L	F/56	R	430	3	8	4.0	2
5L	M/71	R	0	9	9	4.0	6
6L	M/47	L	0	8	5	6.0	10
7R	F/68	L	0	15	14	6.0	4
7L	F/68	L	0	15	14	6.0	4
8R	M/41	L	0	8	11	6.0	2
9L	F/58	L	320	11	9	4.8	2
10L	M/59	L	550	15	6	5.0	2

L, left; R, right; F, female; M, male; LEDD, Levodopa Equivalent Daily Dose.

**Figure 1:**
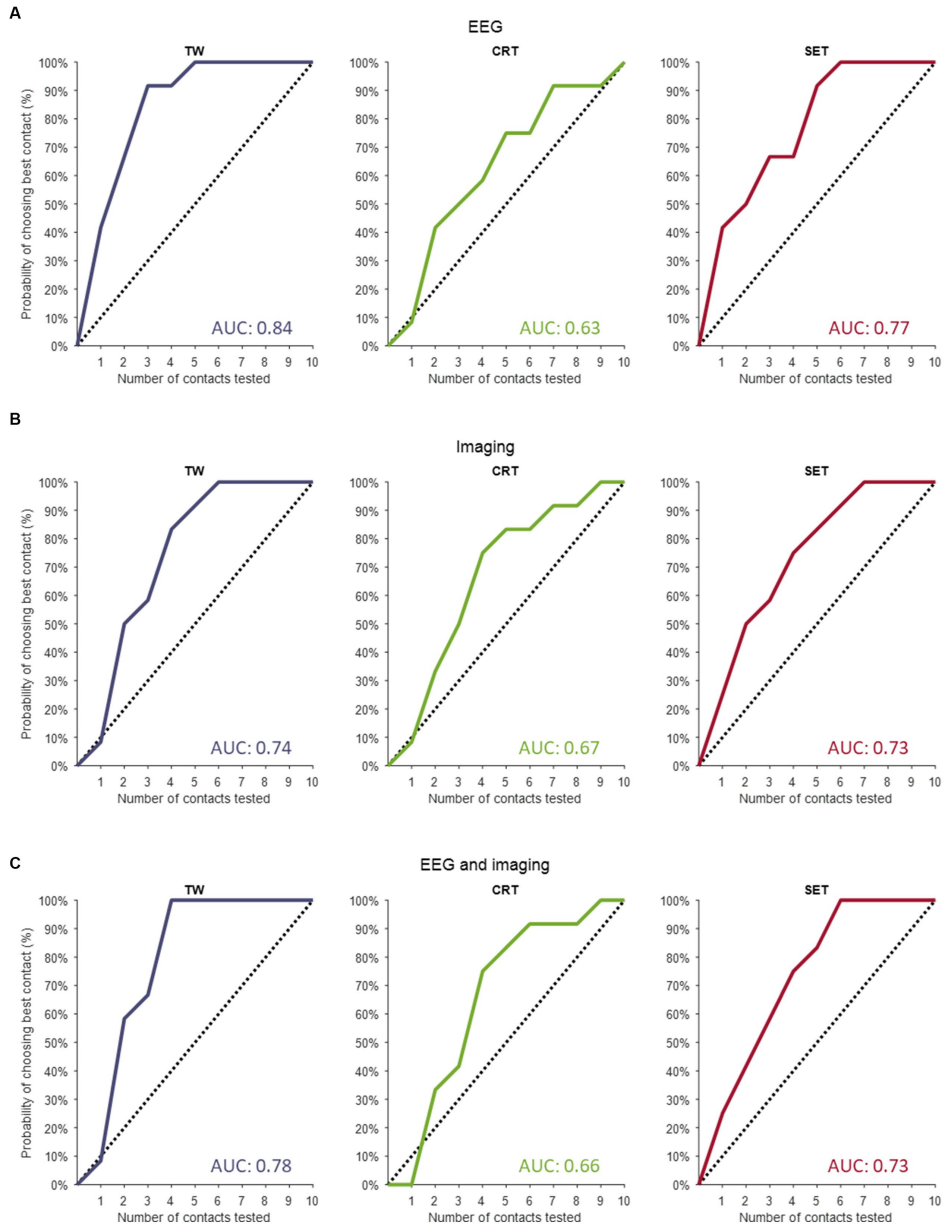
Contact prediction of clinical outcome measures. **(A)** The contact predictions based on the linear mixed models only including EEG data for TW (left panel), for CRT (middle panel) and for SET (right panel). **(B)** The contact predictions based on the linear mixed models only including imaging data for TW (left panel), for CRT (middle panel) and for SET (right panel). **(C)** The contact predictions based on the linear mixed models combining EEG and imaging data for TW (left panel), for CRT (middle panel) and for SET (right panel). The dashed line indicates the chance the programmer has of selecting the best contact if they perform a monopolar review with an increasing amount of randomly selected contacts. The *y*-axis shows the cumulative chance of finding the best contact (in %) as an increasing number of randomly selected contacts are tested (*x*-axis). TW, therapeutic window; CRT, clinical response threshold; SET, side effect threshold.

The predictions resulting from the linear mixed models are illustrated in Figure 1 as the overall probability of identifying the contact with the truly best TW, CRT and SET. When performing a full monopolar review assessment (i.e., eight contacts and two segmented contacts in ring mode), the programmer has a 100% chance of choosing the contact with the truly widest TW, lowest CRT and highest SET (Figure 1, dashed lines). However, when testing three randomly selected contact configurations during a monopolar review, the probability of choosing the contact with the truly widest TW, lowest CRT and highest SET is only 30%. If we performed a monopolar review on the top three contact configurations suggested by the EEG-based prediction models (Figure 1A, solid lines), the probability to find the contact with the truly widest TW increases to 90%, lowest CRT increases to 50% and highest SET increases to 50%. Next, if we performed a monopolar review on the top three contact configurations suggested by the image-based prediction models (Figure 1B, solid lines), the probability to find the contact with the truly widest TW increases to 58%, lowest CRT increases to 50% and highest SET increases to 58%. Lastly, if we performed a monopolar review on the top three contact configurations suggested by the combined prediction models (Figure 1C, solid lines), the probability of finding the contact with the truly widest TW increases to 67%, lowest CRT increases to 42% and highest SET increases to 58%.

The AUC of the dashed line (i.e., chance the programmer has of selecting the best contact configuration if they perform a monopolar review with an increasing number of randomly selected contacts) is always 0.5. The AUCs calculated for the EEG-based, imaging-based and EEG- and imaging-based contact predictions were always greater than 0.5, indicating that all models outperform testing on randomly selected contact configurations (see Figure 1 for AUC values). Next, for TW predictions, we found that the EEG-based model (AUC of 0.84) outperformed the model with only imaging data (AUC of 0.74) and the combined model (AUC of 0.78). Furthermore, for CRT predictions, we found that the imaging model (AUC of 0.67) slightly outperformed the EEG-based model (AUC of 0.63) and the combined model (AUC of 0.66). Lastly, for SET predictions, we found that the EEG-based model (AUC of 0.77) outperformed the imaging model (AUC of 0.73) and the combined model (AUC of 0.73).

**Figure 2:**
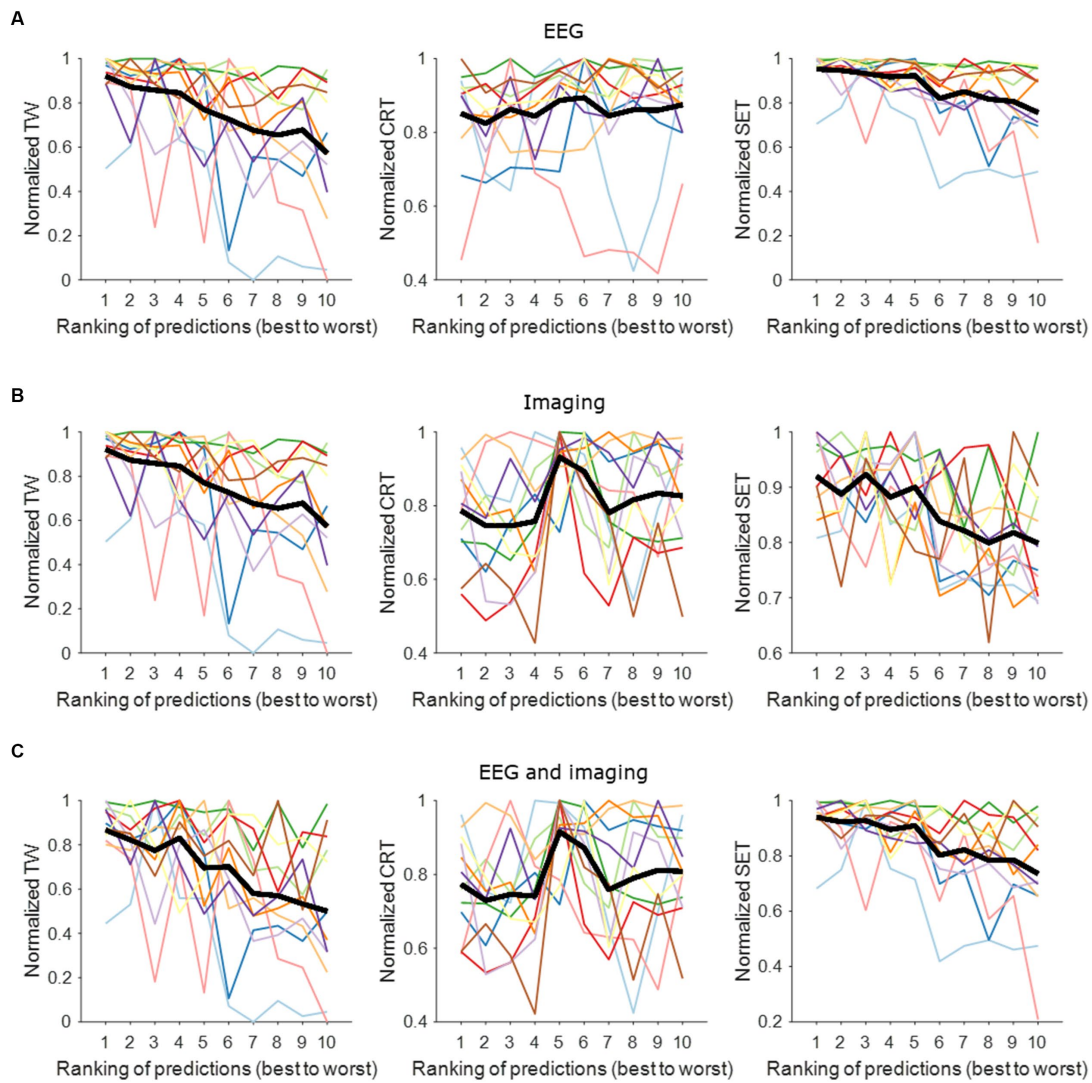
Contact prediction per hemisphere based on EEG data, imaging data, and combined EEG and imaging data. **(A)** EEG-based contact prediction per hemisphere only including data for TW (left panel), for CRT (middle panel) and for SET (right panel). **(B)** Imaging-based contact prediction per hemisphere only including data for TW (left panel), for CRT (middle panel) and for SET (right panel). **(C)** Combined EEG- and imaging-based contact prediction per hemisphere only including data for TW (left panel), for CRT (middle panel) and for SET (right panel). The x-axis shows the ranking of the contacts from best (i.e., widest TW, lowest CRT, highest SET) to worst (i.e., narrowest TW, highest CRT, lowest SET). The *y*-axis shows the normalized stimulation intensity for TW (left panels), SET (middle panels) and CRT (right panels) in mA. The different colors represent the different hemispheres tested. The thick black line represents the average across tested hemispheres. TW, therapeutic window; CRT, clinical response threshold; SET, side effect threshold.

Figure 2 shows the contact performance (in predicting contact with widest TW, lowest CRT and highest SET) ranked from best to worst per hemisphere based on EEG, imaging and a combined model. The normalized mean EEG-based contact prediction indicates that the better ranks predict a wider TW, a low stimulation intensity for CRT and a high stimulation intensity for SET. Similar findings are observed for the imaging-based contact prediction and the combined EEG- and imaging-based contact prediction for the prediction of TW and SET. For CRT, we observe an increase in stimulation intensity between rank 4 and 5. This is because the same CRT value was often noted for multiple contacts in one hemisphere, and they were all assigned a different rank. This resulted in small changes in CRT values predicted by the models and hence, not a straight increasing normalized mean contact prediction using EEG and/or imaging information.

## 4. Discussion

In this study, we used a linear mixed model analysis to investigate if P3 and P10 can be used to predict the best contact to guide DBS programming in PD patients. To investigate the robustness of these models, all electrophysiological data were included in the model, meaning that also data from patients with less apparent P3 and P10 peaks were included in case an EEG experiment would yield mediocre results only. The leave-one-out analysis then provided evidence on how EPs could be used as features for DBS programming.

We found that each contact prediction based on the linear mixed models to find the contact with the widest TW, the lowest CRT and the highest SET including either EEG data or imaging data is superior to the chance the programmer has of selecting the contact with the truly widest TW, lowest CRT and highest SET if they perform a monopolar review with an increasing number of randomly selected contacts. When combining EEG and imaging data in the linear mixed models, we also found that the prediction models are superior to the random selection of contacts in a monopolar review assessment. For SET predictions, the EEG-based model outperformed the imaging-based model and the combined model. We believe that this can be partly explained by the fact that the P10 peak is a very strong peak in amplitude that is highly correlated to side effects as our group published previously (Peeters et al., 2023b), while the P3 peak is lower in amplitude. Hence, these models may be more driven by the larger differences in P10-peaks, resulting in the EEG-based model to outperform the other two models for SET predictions. Next, for CRT predictions, the image-based model outperformed the EEG-based model as well as the combined model. This is most likely due to the fact that the P3 peak, which is positively correlated to therapeutic effects (Peeters et al., 2023b), is rather low in amplitude and hence, the image data may be driving the predictive effect more. Lastly, for TW predictions, the EEG-based model once again outperformed the image-based model and the combined model. This is no surprise as TW is the resulting difference between SET and CRT and SET is more driven by the side effect-related P10 peak.

Programming has become more complicated and time-consuming due to a widened parameter space, the burden on the patient (Ten Brinke et al., 2018; Vitek et al., 2020) which often still leads to suboptimal clinical outcomes in some patients. In this study, we demonstrate that EEG-based biomarkers recorded from the different DBS-contacts separately as well as the segmented contacts in ring mode can be used to create a shortlist (e.g., three contact configurations) of optimal DBS-contacts. This would allow the programmer to focus on fewer but more promising DBS-contacts. As shown here, testing only five contacts of ten configurations of an eight-contact directional lead already yields a 90–100% prediction accuracy (depending on the features added in the model) to find the true contact with the widest TW, thereby expediting the programming time.

Some limitations need to be noted for this study. Firstly, the sample size is modest, though patient programming happens on a patient-specific level. The rather modest sample size could, however, still impact the generalizability of the results described here. Next, we only considered rigidity as the clinical outcome measure used for the monopolar review assessment. The sweet spot atlas (Dembek et al., 2019) used for all imaging analyses in this study, however, considers overall motor improvement using the UPDRS part III. Lastly, the contacts predicted by our novel method provide a strict ranking to approximate the performance of the EEG- and/or imaging-based contact prediction. This means only one contact will be ranked as having the widest TW, the lowest CRT and/or the highest SET. In practice, however, stimulation from more than one contact can result in good clinical outcomes. Thus, a more individualized approach can be applied when using this model for contact prediction.

In previous studies, we reported that EEG-based biomarkers show significant differences in amplitude when stimulating on the different directions using the segmented contacts (Peeters et al., 2023a). Furthermore, we showed that changing the electric field in small incremental steps using MICC (Peeters et al., 2022) results in significant differences in EP amplitudes. Lastly, we also showed that these EEG-based biomarkers may have clinical value (Peeters et al., 2023b). In these previous studies, we showed that P3 was strongest in dorsal contacts closer to dorsal STN (Peeters et al., 2023a,b), but that stimulation from contacts outside of the motor STN border resulted in strong P3 peaks, suggesting the involvement of the zona incerta (ZI) and white matter tracts such as the hyperdirect pathway (HDP). These results thus show therapeutic relevance for the P3 peak. For P10, we find stronger peaks in the more ventral contacts, indicating substantia nigra (SN) involvement and hence, therapeutic relevance as well as these may be relevant for SN-related side effect investigations.

The current study now introduces a practical approach of how EEG-based biomarkers can be used to guide the selection of contacts for testing during DBS programming of PD patients and indicates how well the approach might perform compared to contact testing in a monopolar review assessment. In addition, the current study shows that EEG-based biomarkers can be an alternative or complementary to an existing image-based approach based on an aggregate clinical sweet spot volume and stimulation model (Dembek et al., 2019). A potential advantage of this EEG-based method is that this could potentially be built into the clinician programmer to guide the DBS programming without additional scans or analyses. The programmer could then stimulate each DBS-contact at a fixed stimulation intensity (e.g., similar to impedance measurements at the start of a programming visit) making this a potentially interesting and efficient guidance tool for programming. Furthermore, these EEG-based EPs may be useful in adaptive DBS, a strategy that recently attracted scientific interest (Habets et al., 2018; Guidetti et al., 2021). In theory, adaptive DBS works by responding to input brain signals by providing optimized stimulation parameters to improve the therapeutic efficacy and increase battery longevity. A recent review paper now provided a comprehensive summary of advances for adaptive DBS (Wang et al., 2023), where the researchers suggested that EPs may be a promising source of input signals for adaptive DBS. Obviously, their effectiveness and applicability still need to be confirmed in a large-scale study.

In conclusion, the results shown here indicate that EEG-based biomarkers can be used to guide DBS programming in DBS patients. This EEG-based method could be built into the clinician programmer directly to guide the DBS programming without additional analyses. Ultimately, EEG-based biomarkers can be complementary to existing imaging approaches and can be a valuable contribution to achieving the goal of objective DBS programming for individual patients.

## Data availability statement

The raw data supporting the conclusions of this article will be made available by the authors, upon reasonable request.

## Ethics statement

The studies involving humans were approved by UZ/KU Leuven ethical committee. The studies were conducted in accordance with the local legislation and institutional requirements. The participants provided their written informed consent to participate in this study.

## Author contributions

JP: Conceptualization, Data curation, Formal analysis, Investigation, Methodology, Project administration, Software, Visualization, Writing – original draft, Writing – review & editing. TB: Conceptualization, Data curation, Investigation, Methodology, Validation, Writing – review & editing. AB: Data curation, Investigation, Methodology, Writing – review & editing. TD: Conceptualization, Formal analysis, Methodology, Writing – review & editing. RG: Methodology, Software, Writing – review & editing. JW: Resources, Writing – review & editing. WV: Writing – review & editing. PV: Writing – review & editing. BN: Conceptualization, Methodology, Supervision, Writing – review & editing. MM: Conceptualization, Formal analysis, Funding acquisition, Methodology, Supervision, Validation, Writing – review & editing.

## Footnotes

^1^ www.random.org
